# Dentition Type as a Determinant of Microbial Load Reduction by Antimicrobial Photodynamic Therapy in Deep Dentin Caries: A Systematic Review and Meta-analysis

**DOI:** 10.30476/dentjods.2023.98616.2098

**Published:** 2024-12-01

**Authors:** Zahra Baghani, Malihe Karrabi, Hossein Assarzadeh

**Affiliations:** 1 Dept. of Periodontics, Faculty of Dentistry, Sabzevar University of Medical Sciences, Sabzevar, Iran; 2 Dept. of Prosthodotics, Faculty of Dentistry Sabzevar University of Medical Sciences, Sabzevar, Iran

**Keywords:** Deep carious lesions, Photochemotherapy, Dentition, Permanent, Bacterial Load, Lactobacillus

## Abstract

**Statement of the Problem:**

Antimicrobial photodynamic therapy (aPDT) is a protocol proposed for reduction of bacterial load in deep dentin caries in primary and permanent dentitions. However, considering the difference in the morphology of dentinal tubules in primary and permanent teeth, the effect of this treatment may be different on the two dentition types.

**Purpose:**

This systematic review and meta-analysis aimed to assess the effect of type of dentition as a determinant of microbial load reduction by aPDT in deep dentin caries.

**Materials and Method:**

An electronic search was conducted in Scopus, Web of Science, Cochrane, Medline, and Embase databases, from the first record until April 30, 2022. After article screening by three reviewers, seven studies were included in this meta-analysis. The mean log of *Streptococcus mutans* (*S. mutans*) count, *Lactobacillus spp.* count, and the entire bacteria in the cavity before and after aPDT was calculated with 95% confidence interval (CI), and compared between the two groups of primary and permanent teeth by the random effect model. The I^2^ test was applied to assess the heterogeneity of the findings. Publication bias was evaluated by visual examination of the funnel-plot symmetry

**Results:**

Of 7 retrieved articles, analysis of 3 studies on permanent teeth showed that aPDT caused a significant reduction in total bacterial count in the cavity [SMD: 0.64, 95% CI:(0.31, 0.96), *p*= 0.0001), *S. mutans* count [SMD: 0.92, 95% CI:(0.58, 1.25), *p*< 0.0001], and *Lactobacillus spp.* [SMD: 1.1, 95% CI:(0.76, 1.45), *p*< 0.00001)]. Analysis of the remaining 4 studies on primary teeth indicated that aPDT had a significant effect only on *S. mutans* count [SMD: 0.60, 95% CI:(0.23, 0.97), *p*= 0.001), and its effect on total bacterial count of the cavity [SMD: 0. 90, 95% CI:(-0.02, 1.82), *p*= 0.05] and *Lactobacillus spp.* [SMD: 0.18, 95% CI:(-0.29, 0.64), *p*= 0.45)] was not significant.

**Conclusion:**

The results showed that aPDT could serve as an effective adjunct for reduction of microbial load in permanent teeth.

## Introduction

Dental caries is a biofilm-related infectious disease that causes the destruction of the tooth structure. *Streptococcus mutans* (*S. mutans*) is the most important microorganism in dental biofilm, which adheres to the enamel surface, and initiates the process of caries development by acid production. Other microorganisms such as *Streptococcus sobrinus*, *Streptococcus gordonii*, *Lactobacillus spp.*, and *Actinomyces species* are involved in continuation of this process and progression of caries [ 1 - 2 ]. Therefore, elimination of the abovementioned microorganisms and their toxins is a fundamental step that increases the clinical service of restorations. Nonetheless, complete caries removal in deep parts of the cavities is often challenging because it may cause pulp exposure especially in young patients. On the other hand, leaving the bacteria in deep areas can result in treatment failure due to caries recurrence [ 3 - 6 ]. 

According to the commonly practiced treatment protocols, the first suggested strategy to overcome this problem is to apply antibacterial agents in the cavity. However, it should be noted that overuse of antibiotics can lead to emergence of resistant species [ 7 ] Antimicrobial photodynamic therapy (aPDT) is another protocol proposed for reduction of bacterial load while preventing pulp exposure in deep cavities. This two-step process, which includes the use of a photosensitizer (PS) and its irradiation with appropriate wavelength of light, generates reactive oxygen species and free radicals that damage the microorganism components, irreversibly change the metabolic activity of the bacteria, and eventually lead to the death of microorganisms in the cavity [ 8 - 12 ]. 

Recent randomized clinical trials (RCTs) compared the effects of aPDT on microorganisms in deep carious lesions by comparing their count before and after treatment, and reported contradictory results in primary teeth, permanent teeth, or a combination of them [ 13 - 17 ]. It should be noted that the bacterial flora of primary and permanent dentitions is different in both sound and carious teeth [ 18 ]. Moreover, the diversity of bacterial species involved in permanent tooth caries is much higher than that in primary teeth. In addition, *S. mutans* has a more prominent role in deep caries in primary teeth, compared with permanent teeth. Approximately 10% to 15% of adults with active caries do not have a detectable level of *S. mutans*, indicating the involvement of other acidogenic bacteria [ 18 ]. Therefore, it appears that bacterial diversity, in addition to other parameters such as differences in laser parameters and type of PS, may be responsible for different outcomes of aPDT in primary and permanent teeth. 

A previous meta-analysis focused on the effects of aPDT on deep caries in both the primary and permanent teeth, and indicated its positive effect on the total bacterial count in the cavity, and the most important microorganisms involved in caries process (i.e. *S. mutans* and *Lactobacillus spp.*) [ 19 ]. However, it appears that not differentiating between the primary and permanent dentitions and methodological flaws can lead to high heterogeneity and bias, and compromise the reliability of the results. Therefore, ambiguities still remain regarding the above-mentioned topics, which need to be elucidated. Accordingly, this study aimed to assess the effect of type of dentition as a determinant of microbial load reduction by aPDT in deep dentin caries.

## Materials and Method

### PICO Protocol and Registration

This systematic review and meta-analysis was conducted according to the PRISMA statement, Cochrane Collaboration [ 20 ] and Systematic Review Checklists. The review protocol was published in PROSPERO, the Prospective Register of Systematic Reviews (ref CRD4202 2323078). The inclusion criteria according to the population, intervention, comparisons and outcomes (PICOS) were as follows:

Population (P): Healthy children or adults with deep caries in primary or permanent teeth, and no intake of medications that would affect the study results.

Intervention (I): Application of aPDT for elimination of bacteria and reduction of bacterial load Comparison (C): One group treated with aPDT and one group treated without aPDT, and/or comparison of bacterial load before and after aPDT. 

Outcome (O): Change in count of *S. mutans*, *Lactobacillus spp.*, and the entire bacteria in the cavity before and after aPDT.

Study design (S): RCTs

### Focused Question

The focused clinical question of this study was that “whether type of dentition (primary/permanent) can serve as a determinant of the efficacy of aPDT for reduction of bacterial load in deep primary and permanent caries”. 

### Search strategy

An electronic search was conducted using a combination of MeSH terms and text words as follows:

“Photochemotherapy” (MeSH) OR “Photodynamic therapy” 

AND “Dental caries” (MeSH) OR “Carious dentin” OR “Dentin caries” OR “Dentinal caries”.

**Figure 1:**
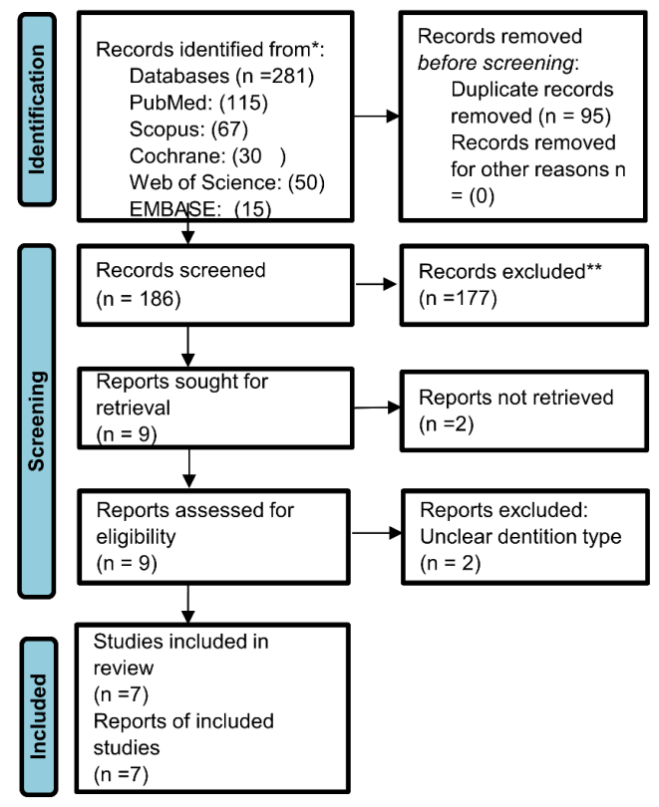
Flowchart of search strategy

Two masked researchers (ZB and MK) searched the MEDLINE via PubMed, Cochrane and Embase, Scopus, and Web of Science databases for articles published until April 30, 2022. An inter-reviewer reliability analysis was also carried out between the two researchers, and disagreements regarding the inclusion criteria were resolved by discussing the third author (HA). Next, the full texts of the eligible articles were read. Figure 1 shows the PRISMA flow diagram of the screening process. 

### Inclusion and Exclusion Criteria

The inclusion criteria were the RCTs articles that used aPDT for reduction of bacterial load in deep caries according to dentition type (primary/permanent). In addition, the results of English language studies were reported as mean±SD of bacterial count in the cavity before and after aPDT application, and there was no limitation with respect to the type of PS, or voltage, or wavelength of light.

Studies that did not mention the type of dentition and did not report the number of microorganisms before and after aPDT or their difference were excluded. *In vitro* studies, review articles, case reports, unpublished studies, letters to editors, and abstracts were not included in this meta-analysis, too. 

### Data Extraction

To perform a meta-analysis in the present study, the bacterial count before and after aPDT was evaluated in the included studies. After reviewing the full-text of the articles, qualitative analysis and data extraction were carried out by two independent reviewers (ZB and MK). The second author (MK) confirmed the accuracy of the extracted data, and ambiguities were discussed with the third author (HA) until a consensus was reached. Finally, the following information was extracted:

Study title (along with the name of the first author), publication year, number of participants, dentition system, microbial load (mean ± standard deviation), details of the intervention such as laser wavelength (nm), duration of irradiation, light source, PS type, laser output, energy density (J ∕cm^2^) and spot size (mm^2^) (Table 1). 

**Table 1:** Descriptive summary of the included studies

	Study	Country	Number of participants	Age of participants	Wave length (nm)	Time	Light source	Dose (J/cm^2^)	Output Power (mW)	Spot (mm^2^)	Ps type	Dentition system
1	Borges *et al*. [ 7]	Brazil	5	19-36	620 to 660 predominant wavelength of 638.8	600s	Red light-emitting diode	94	40	9.5	TB	Permanent
2	Guglielmi *et al*. [ 3]	Brazil	26	8-25	660	90s	InGaAIP	320	100	2.8	MB	Permanent
3	Melo *et al*. [ 24]	Brazil	45	<18	630	WD	WD	94	150	6 mm	MB	Permanent
4.1	Oliveira *et al*. [ 25]	Brazil	10	5-7	600	90s	Red low power laser light source	320	100	WD	MB	Primary
4.2	Oliveira *et al*. [ 25]	Brazil	10	5-7	630	60s	Red LED light source	30	100	WD	TB	Primary
5	Neves *et al*. [ 14]	Brazil	19	6-10	660	120s	InGaAlP	120	100	4	MB	Primary
6	Ornellas *et al*. [ 11]	Brazil	18	4-11	660	90s	InGaAIP	300	100	3 mm²	MB	Primary
7	Alves *et al*. [ 15]	Brazil	20	6-8	660	180s	InGaAlP	640	100	WD	MB	Primary

Note: MB: methylene blue; TBO: toluidine blue; aPDT: antimicrobial photodynamic therapy; WD: without data

### Quality Assessment and Risk of Bias

According to the Cochrane Handbook for Systematic Reviews of Interventions, the Revised Cochrane Risk of Bias (RoB) tool for randomized trials, version 2.0 (RoB 2) was independently assessed for each included study by two of the authors (ZB and MK). 

Risk of bias was categorized as low, some concerns, and high. Disagreements between the reviewers were resolved by consultation with the third author (HA).

### Data Synthesis and Statistical Analysis

Details of the studies that were independently extracted by two reviewers (ZB and MK) were as follows: Type of dentition, number of participants, and count of *S. mutans*, *Lactobacillus spp.*, and the entire bacteria present in the cavity before and after aPDT, or their difference, reported as mean and standard deviation. The extracted data were tabulated and transferred to RevMan version 5.0. There was no missing data that would necessitate contacting the respective authors. 

The articles were categorized based on the type of dentition evaluated (primary/permanent). The effect of treatment was reported as the mean difference with 95% confidence interval (CI). 

The random-effect model of RevMan version 5 was used for data analysis at *p*< 0.05 level of significance. To detect statistical heterogeneity, the forest plots were visually inspected through the presence of outlier studies. To assess the heterogeneity of the findings, the I^2^ test was applied in the range of 0-100% with the following explanation: 

0% = no evidence of heterogeneity, 30-60% = moderate heterogeneity, and 75-100% = high heterogeneity [ 21 ]. In order to assess the outcomes after negation of heterogeneous studies, a sensitivity analysis was performed [ 22 ]. Also, the publication bias was analyzed by visual assessment of the funnel plot symmetry [ 23 ].

## Results

### Selection of Studies

Figure 1 indicates the PRISMA flowchart of article selection. Electronic search of Medline (149), Cochrane (30), Embase (15), Scopus (67) and Web of Science (50) databases yielded a total of 281 articles. After omitting duplicates, 186 articles remained. Nine articles remained after screening of the titles and abstracts (inter-reviewer agreement: kappa=0.9) that met the inclusion criteria, and underwent full-text analysis [ 3 , 7 , 11 , 14 - 17 , 24 - 25 ]. In full-text analysis, 2 articles were excluded since they did not mention the type of dentition dentition [ 16 - 17 ] (Table 2). 

**Table 2:** List of reasons for exclusion of articles in the stage of full text assessment

Excluded study	Reasons
Araujo *et.al.* [ 16]	Unclear dentition type
Longo *et.al.* [ 17]	Unclear dentition type

Finally, data from 7 articles (inter-reviewer agreement kappa=1) were extracted and underwent qualitative and quantitative analyses by the software [ 3 , 7 , 11 , 14 - 15 , 24 - 25 ].

### General Characteristics of the Included Studies

A total of 7 RCTs were evaluated in this study; out of which, 4 had been conducted on primary dentition [ 11 , 14 - 15 , 25 ] and 3 had been conducted on permanent dentition [ 3 , 7 , 24]. The number of teeth evaluated in the studies ranged from 5 to 45 teeth. The age range of patients with primary dentition was 4-11 years while the age range of patients with permanent dentition was 8-36 years. In 5 studies, the teeth were divided into two groups of test (incomplete caries removal + aPDT) and control (incomplete caries removal without aPDT), and the bacterial count was compared between the test and control groups [ 7 , 14 - 15 , 24 - 25 ]. In two studies, aPDT was performed after incomplete caries removal and counting of the bacterial count in the cavities, and sampling from the cavities was performed again after aPDT such that the change in bacterial count after aPDT compared with baseline was the criterion to assess the efficacy of aPDT [ 3 , 11 ] (Figure 1).

### Characteristics of the included datasets

### Laser Parameters in the Included Studies

InGaAlP laser was used in 4 studies [ 3 , 11 , 14 - 15 ] while Borges *et al*. [ 7 ] used red light emitting diode (LED) and Oliveira *et al*. [ 25 ] used red low power laser along with methylene blue (MB) and red LED with toluidine blue (TB). The abovementioned lasers were irradiated on the tooth surface with the wavelength range of 620 to 660 nm with irradiation period of 60 to 600 seconds, energy density of 30 to 640 J/cm^2^, and output power of 40 to 150 mW. The type of light source and its application time were not mentioned in one study [ 24]. The spot size of laser ranged from 2.8 to 9.5mm^2^ [ 3 , 7 , 11 - 14 , 24 ]. Two studies did not report the spot size [ 15 , 25 ]. MB was the most commonly used PS in the reviewed studies [ 3 , 11 , 14 - 15 , 24 - 25 ], while, TB was only used by Borges *et al*. *et al*. [ 7 ], and in one group in the study by Oliveira *et al*. [ 25]. 

### Risk of bias in individual studies

**Figure 2:**
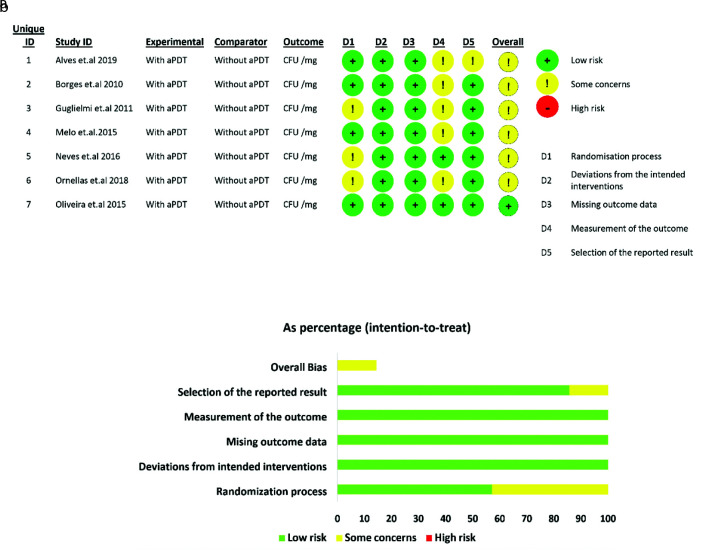
Risk of bias assessment summary of the included studies based on the consensual answers of two independent assessors **a:** MK and ZB), **b:** Risk of bias graph

Two reviewers (MK and ZB) according to the recommendations of the CONSORT statement using ROB-2 tool [ 26 ] independently calculated risk of bias for each study (Figure 2).

In this process, as shown in Figure 2a, 6 studies did not have one or more parameters required for quality assessment, and were categorized under the category of “some concerns”. One study m*et al*l the criteria and had a low risk of bias. Randomization of samples was reported in 4 studies, using different randomization tools such as using a computer spreadsheet [ 15 , 11 ] or a computer-generated list [ 24 ]. Neves *et al*. [ 14 ] did not mention anything about randomization. In two studies, randomization was not performed since aPDT was performed for all participants, and the bacterial count was measured before and after aPDT in all cases [ 3 , 11 ]. 

Samples were evaluated by a blind examiner in two studies [ 14 , 25 ], while; in the study by Melo *et al*. [ 24 ], only the participants were blind. In studies by Ornellas *et al*. [ 11 ], and Guglielmi *et al*. [ 3 ], the assessors were not blind since all participants received the same intervention. Two other studies did not mention anything about blinding of the assessor(s) [ 24 , 15 ]. 

### Meta –analysis and Sensitivity analysis

**Figure 3:**
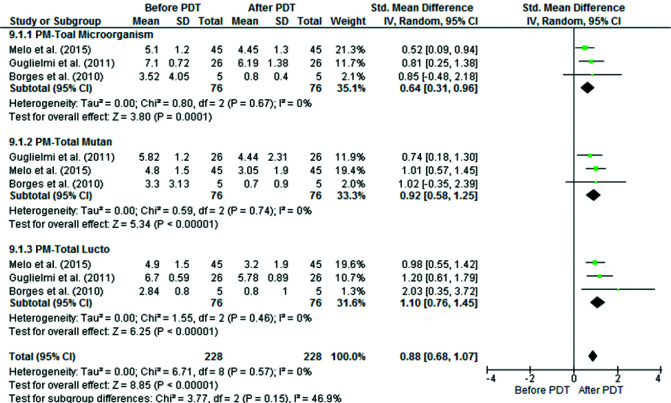
Comparison of the mean and standard deviation of log10 colony forming units per milliliter (CFUs/mL) before and after aPDT in three subgroups of **a:** all viable bacteria, **b:** *S. mutans*, and **c:** *Lactobacillus spp.* in permanent teeth

This meta-analysis included parallel-design RCTs on the efficacy of aPDT by comparing the microbial load in deep caries of primary and permanent teeth. The results of the forest plots showed that aPDT significantly decreased the bacterial count in permanent tooth caries and this effect was significant in all three subgroups of all bacteria [SMD: 0.64, 95% CI: (0.31, 0.96), *p*= 0.0001], *S. mutans* [SMD: 0.92, 95% CI: (0.58, 1.25), *p*< 0.0001] and *Lactobacillus spp.* [SMD: 1.1, 95% CI: (0.76, 1.45), *p*< 0.00001] (Figure 3).

**Figure 4:**
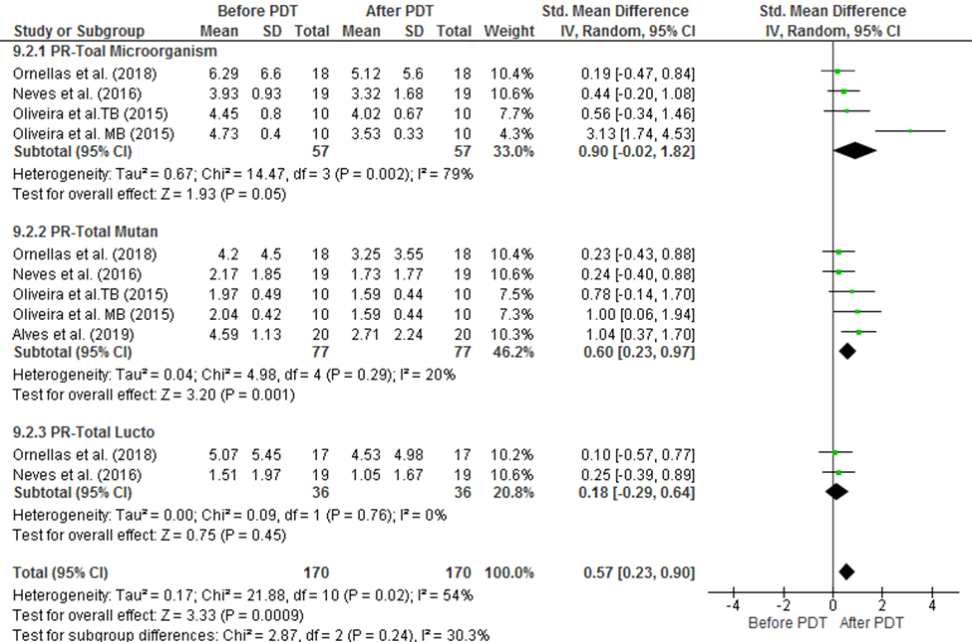
Comparison of the mean and standard deviation of log 10 CFUs/mL before and after aPDT in three subgroups of **a:** all viable bacteria, **b:** *S. mutans*, and **c:** *Lactobacillus spp.* in permanent teeth

However, in primary teeth, aPDT only caused a significant reduction in *S. mutans* count [SMD: 0.60, 95% CI: (0.23, 0.97), *p*= 0.001] and its effect on *Lactobacillus spp.* [SMD: 0.18, 95% CI: (-0.29, 0.64), *p*= 0.45] and the entire microorganisms in the cavity [SMD: 0.90, 95% CI: (-0.02, 1.82), *p*= 0.05] was not significant (Figure 4). 

**Figure 5:**
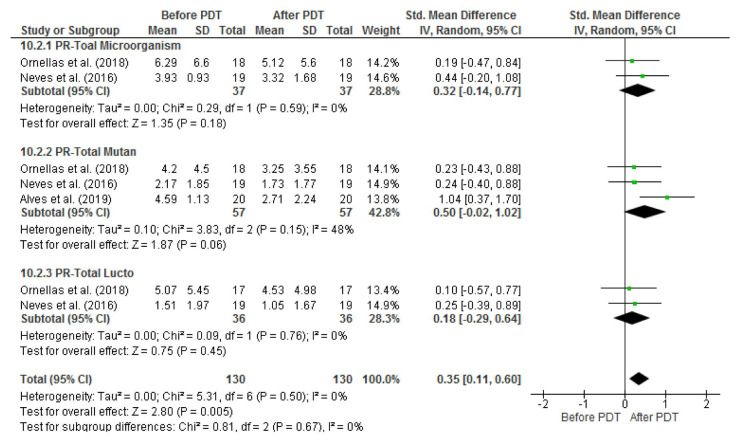
Forest plots based on sensitivity analysis showing the overall CFUs/mL reduction without the outlier study

The degree of heterogeneity was low in studies conducted on permanent teeth; however, considering the high heterogeneity of studies conducted on primary teeth, one outlier study was excluded by visual inspection the study by Oliveira *et al*., [ 27 ], and sensitivity analysis was conducted on the remaining studies [ 3 , 8 , 14 - 15 , 11 , 24]. By elimination of the outlier study, the results were not significant for the entire bacteria in the cavity [SMD: 0.32, 95% CI: (-0.14, 0.77), *p*= 0.18], *S. mutans* [SMD: 0.50, 95% CI: (- 0.02, 1.02), *p*= 0.06], and Lactobacillus spp. [SMD: 0.18, 95% CI: (-0.29, 0.64), *p*= 0.45] with low heterogeneity T2 = 0.00; X2 = 5.31(*p*= 0.50); I2 = 0%] (Figure 5). 

### Publication bias

**Figure 6:**
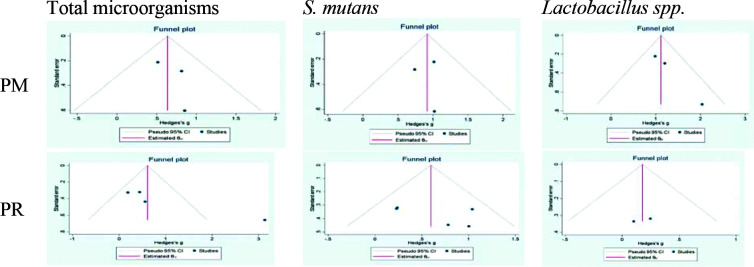
Funnel plots for log CFUs/mL reduction adjusted with Trim and Fill method for primary (PR) and permanent (PM) dentition studies. Circles indicate the included studies (STATA Software)

**Figure 7:**
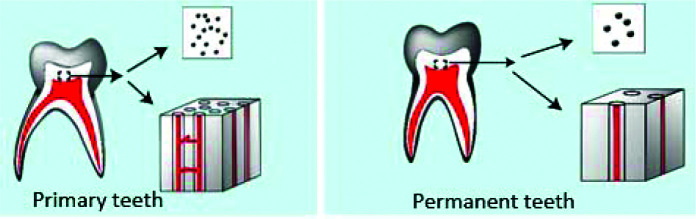
Dentinal tubules density in primary teeth versus permanent teeth

**Figure 8:**
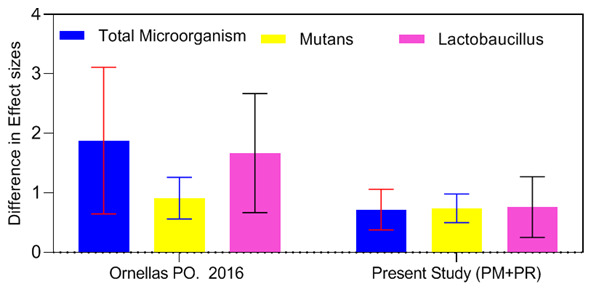
Effect size comparison of the present study with the meta-analysis by Ornella *et al*. [ 19] regarding the effect of aPDT on microbial load

**Figure 9:**
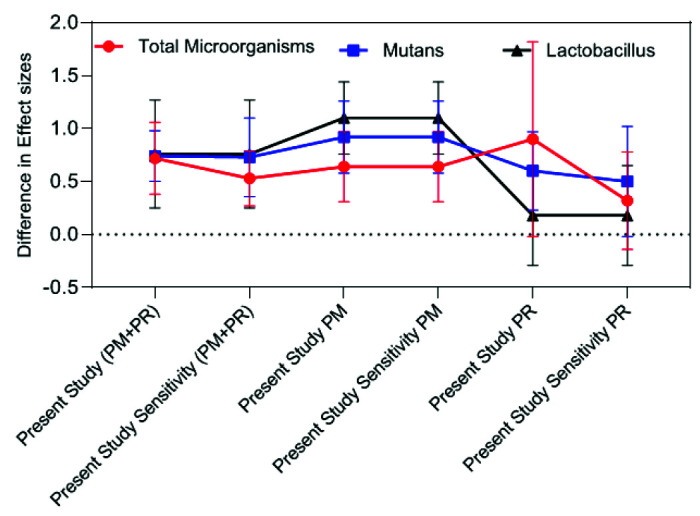
Effect size comparison of the count of different microorganisms in primary dentition, permanent dentition, and primary dentition + permanent dentition

The forest plot of the studies conducted on primary and permanent teeth was drawn using STATA version 16 software (STATA Co., College Station, TX, USA). The results indicated absence of asymmetry in studies assessing the cariogenic microorganisms in primary and permanent dentitions (Figure 6). This analysis was then conducted on a combination of primary and permanent teeth, and showed no asymmetry (Figures 7-9).

## Discussion

The results of the present meta-analysis revealed that aPDT significantly decreased the count of cariogenic microorganisms (*S. mutans* and *Lactobacillus spp.*) in deep cavities in permanent teeth, but its effect on the count of cariogenic bacteria in primary teeth was not significant. Analysis of the data in the present study was conducted in three subgroups of entire bacterial count in the cavity, *S. mutans* count, and Lactobacillus spp. count. The results showed significant efficacy of aPDT for reduction of all three subgroups in permanent teeth.

However, in primary dentition, aPDT only caused a significant reduction in *S. mutans* count, and its effect on the entire bacterial count in the cavity and Lactobacillus spp. was not significant. Thus, in response to the focused question of the study “whether type of dentition (primary/permanent) can serve as a determinant of the efficacy of aPDT for reduction of bacterial load in deep primary and permanent caries”, it may be stated that irrespective of the adopted laser parameters, type of dentition is a key factor in the efficacy of aPDT. 

Some recent review studies [ 27 - 28 ] on the efficacy of aPDT as an adjunct for treatment of deep carious lesions, tooth preparation, and endodontic debridement supported the application of aPDT to improve the anti-bacterial efficacy of restorative and endodontic procedures in deciduous and permanent teeth. In addition, their results regarding the efficacy of aPDT in minimizing the count of cariogenic bacteria in primary dentin are debatable due to lack of long-term clinical trials and robust study designs. In all of the above-mentioned studies, the philosophy behind the use of aPDT in deep caries was to clean and disinfect the cavity and decrease the bacterial load without causing bacterial resistance [ 7 ]. In fact, aPDT quickly eliminates the microorganisms within a couple of minutes, and can decrease the risk of pulp exposure, and increase the likelihood of arresting the carious lesion without invading or removing the adjacent tooth structure [ 29 ]. The photosensitization mechanism in aPDT is initiated by light irradiation and its absorption by the PS. Next, the obtained energy is spent to produce oxygen free radicals and singlet oxygen (-1O2) [ 16 , 30 ], which cause inactivation of the adjacent bacteria locally by induction of oxidative reactions in the cell wall, lipid membrane, enzymes, and nucleic acids of microorganisms [ 17 ]. The above-mentioned oxidative reactions cause the death of microorganisms. Three mechanisms such as DNA damage, damaging the cytoplasmic membrane and subsequent leakage of cell components or inactivation of the membrane transport system, and inactivation of proteins and cellular enzymes are proposed in this process [ 31 ].

Considering its mechanism of action, it is evident that aPDT is more effective in inactivation of Gram-positive bacteria due to their unique cell wall structure and possession of a thick external peptidoglycan layer, compared with Gram-negative bacteria [ 32 ]. In this process, *S. mutans* is inactivated mainly through the membrane damage as the result of lipid peroxidation [ 33 ]. On the other hand, according to the findings of a previous study on microbial flora of sound and carious primary and permanent teeth, the bacterial flora of primary and permanent dentitions is not the same, and the count of *S. mutans* and *Lactobacillus spp.* is higher in carious primary teeth than permanent teeth. Moreover, the ratio of Gram-positive to Gram-negative bacteria is higher in primary teeth than permanent teeth [ 18 ]. Thus, since aPDT is more effective on Gram-positive bacteria, it is expected to be more effective on primary teeth; however, the present results proved otherwise, and showed that aPDT was more effective on permanent teeth. Such an unexpected result may be due to the fact that the free radicals generated during PS application and its laser irradiation in primary carious lesions do not have adequate access to the existing bacteria, as they do in permanent teeth, due to different structure of primary and permanent dentitions. Evidence shows that the density of dentinal tubules in primary teeth is 3 times higher than that in permanent teeth [ 34 ]. Since open and wide dentinal tubules in areas close to the pulp chamber (with a diameter > 4µm) are suitable for accumulation and lodging of oral streptococci (with 0.5-0.7µm diameter) [ 35 ], the 3 times higher density of dentinal tubules in primary teeth (Figure 7), along with their straighter path, and higher number of canaliculi between the tubules result in higher permeability of primary dentin to bacteria and their toxins, providing a suitable shelter for them and protecting them against oxygen free radicals [ 34 , 36 - 37 ]; whereas, PSs such as MB and TB can only penetrate by 45–60µm (average 52.6µm) and 190 μm, respectively, into the carious dentin since the altered structure of carious dentin does not allow their sufficient penetration [ 3 , 38 - 40 ] (Figure 7).

The generated free radicals cannot penetrate deep into dentinal tubules either due to their short half-life (∼10–320 ns), limiting their diffusion to approximate depth of only 10–55 nm [ 41 - 45 ]. Although oral streptococci can be found up to a depth of approximately 200 µm in sound dentin, even this depth of penetration can be considerably higher in carious dentin [ 35 , 46 ]. Thus, the bacteria lodged in deeper areas are not accessible by the free radicals and survive aPDT. Moreover, it is noteworthy that sample collection from carious dentin is performed by hand instruments, which undoubtedly access deeper parts of dentin. Therefore, samples are collected from the tubules filled with bacteria. However, in sampling from permanent teeth, which includes participants with a wide age range, dentinal tubules have a lower density, have a curved S-shaped path, and do not have canaliculi in inter-tubular spaces. In addition, factors such as occlusal contacts, attrition, and trauma from occlusion can cause secondary and reparative (tertiary) dentin deposition, which would cause a reduction in diameter of dentinal tubules and decrease their permeability [ 47 - 49 ]. Therefore, bacteria and their toxins have a lower chance of penetrating deep into dentin and take refuge against free radicals. Resultantly, free radicals can effectively eliminate the bacteria present in superficial dentin. 

Another finding than can confirm the aforementioned statements is that although *S. mutans* count significantly decreased in both the primary and permanent teeth in the present study, its reduction was significantly greater in permanent teeth (*p*< 0.0001) than primary teeth (*p*= 0.001). Even after elimination of the outlier study that caused high heterogeneity in primary teeth, the results regarding *S. mutans* were not significant in this dentition, whereas, considering the higher percentage of *S. mutans* in primary teeth [ 18 ], a greater reduction in *S. mutans* count was expected in primary dentition. Therefore, it may be assumed that the density and form of dentinal tubules are important factors affecting the efficacy of aPDT. In other words, different structure and form of dentinal tubules in primary teeth, which are responsible for faster caries progression in this dentition [ 36 ], can also be responsible for lower efficacy of aPDT in the primary dentition. 

It should be noted that in addition to not achieving the desired result following aPDT in primary teeth, allocation of 5 minutes of time for the effect of PS to take place, and 90 to 180 seconds of laser irradiation could decrease the cooperation of pediatric patients in the rest of the procedure. In addition, in biomimetic adhesive dentistry, a minimum of 5 minutes has been recommended for maturation of the hybrid layer by decoupling with time [ 50 - 51 ]. Thus, addition of another 6.5 to 8 minutes to the abovementioned time period can compromise the cooperation of children, especially in final stages of restoration, and eventually result in a restoration with inadequate coronal seal and suboptimal standards due to hydrolysis of the resin-dentin interface. Obviously, provision of a hermetic coronal seal has a much more important role in durability and success of restorations than disinfection of deep dentin.

A noteworthy issue is that in aPDT, blue-type PSs (TB and MB) should only be used with lasers in 635-660nm wavelengths, because in wavelengths higher than 800 nm (infrared), no photodynamic reaction occurs with these PSs [ 52 ]. 

Assessment of the efficacy of aPDT for reduction of bacterial load and comparison of its effect size in different meta-analyses can greatly help in selection of a successful treatment plan. As reported in the results section, the effect of aPDT on the entire bacterial count in the cavity, and Lactobacillus spp. in the present study was approximately 50% lower than that reported by Ornellas *et al*. [ 19 ], while the difference between the results of the two studies was smaller for the effect of aPDT on *S. mutans* count. Since the effect of confounding factors was not adjusted, and the primary and permanent dentitions were not independently assessed in the meta-analysis by Ornellas *et al*. [ 19 ], the two dentition systems could not be separately analyzed, and thus, these results are not 100% reliable (Figure 8). 

 In comparison of the effect size obtained from the present meta-analysis regarding different microorganisms in primary and permanent dentitions, it was noticed that following aPDT, the reduction in total bacterial count in the cavity in primary teeth was greater than that in permanent teeth; while, the results were different regarding *S. mutans* and Lactobacillus spp. count, and aPDT caused a greater reduction in their count in permanent teeth, such that it may be even stated that aPDT had no significant effect on Lactobacillus spp. count in primary teeth. Comparison of the results after eliminating the outlier study indicated a significant reduction in total microbial count of the cavity in primary dentition; the magnitude of change was insignificant for *S. mutans*, while this effect remained unchanged on *Lactobacillus spp.* due to absence of any outlier study. Comparison of the effect size obtained from sensitivity analysis in permanent dentition revealed no change due to absence of any outlier study (Figure 9). 

The results of this study are important from the point of view that unnecessary treatments for the primary dentition are avoided. However, in studies included in this meta-analysis, the teeth were divided into two groups of primary and permanent, and the effect of age, which is a fundamental factor in change in dentinal tubules, was not considered. Since change in structure of dentinal tubules is a factor that depends on age, and environmental conditions, permanent teeth may respond differently to this treatment at different ages. Also, presence of confounding factors such as variations in dosage (energy density), duration of radiation, type of PS, adopted light source, and spot size in different studies can cause heterogeneity in the results, such that that the use of doses above 10 J/cm^2^ for wounds would be controversial. However, in clinical practice, doses from 10 to 30 J/cm^2^ are used especially for chronic wounds. Such a high energy density can lead to unwanted photo-thermal effects and tissue damage [ 53 - 54 ]. Moreover, the antibacterial effect of PDT by laser or LED is controversial [ 55 - 56 ]. It appears that optimization of appropriate wavelengths of LED is the key to this problem which should be further assessed [ 57 ].Therefore, more precise studies on the application of aPDT in clinical procedures with similar methodology are required to obtain more reliable results. 

## Conclusion

Analysis of the reviewed studies revealed that aPDT can significantly decrease the load of microorganisms involved in permanent tooth caries, but this effect was not significant on primary tooth caries. Despite the limitations of the present meta-analysis, it indicated that aPDT could be used as an effective adjunct for reduction of microbial load in deep dentin caries in permanent teeth. However, the efficacy of its application for primary teeth still remains questionable. 
